# The EQ-5D-3L valuation study for Bermuda: using an on-line EQ-VT protocol

**DOI:** 10.1007/s10198-024-01701-2

**Published:** 2024-07-09

**Authors:** Henry Bailey, Bram Roudijk, Ricky Brathwaite

**Affiliations:** 1 Department of Economics, The University of the West Indies, St Augustine Campus, St. Augustine, Trinidad and Tobago; 2 HEU, Centre for Health Economics, The University of the West Indies, St Augustine Campus, St. Augustine, Trinidad and Tobago; 3 EuroQol Research Foundation, Rotterdam, The Netherlands; 4 Department of Psychiatry, Erasmus University Medical Center, Rotterdam, The Netherlands; 5 Bermuda Health Council, Health Economics Directorate, Hamilton, Bermuda

**Keywords:** EQ-5D-3L, EQ-5D-5L, Bermuda, Discrete choice experiment, Composite time trade off, I19

## Abstract

**Background:**

In many countries, methods of economic evaluation and Health Technology Assessment are used to inform healthcare resource allocation decisions. These approaches often require EQ-5D health outcomes measures. This study aimed to create an EQ-5D-3L value set for Bermuda from which EQ-5D-5L Crosswalk values could be obtained.

**Methods:**

Respondents in Bermuda were recruited locally. A team of Trinidad-based interviewers with prior EQ-5D-3L valuation experience conducted valuation interviews on-line using the EQ-VT protocol. Respondents completed composite time-trade off (cTTO) and discrete choice experiment (DCE) tasks. A hybrid model that included both the cTTO and DCE data was estimated. An EQ-5D-5L crosswalk value set was then created from the EQ-5D-3L index values. Coefficients in the resulting crosswalk model were compared with those of crosswalk and valuation studies from other countries.

**Results:**

The valuation tasks were completed by a near-representative sample of 366 adult Bermuda citizens. Half of the respondents reported being in state 11111. The lowest EQ VAS and EQ-5D-3L index values were 20 and – 0.120 respectively. The hybrid model produced all logically consistent and statistically significant coefficients that in turn produced index values that were very similar to those obtained in a preliminary model (MAD of 0.027).

**Discussion:**

The on-line EQ-VT valuation study was successfully conducted in Bermuda and the values therein can now be used for economic analysis in Bermuda. The Bermuda values differed considerably from those of the other countries against which they were compared. Challenges were encountered with recruitment for an on-line survey in a small population.

## Introduction

All health systems (large and small) face challenges of allocating scarce resources [1]. As epidemiological trends and available technologies develop, these challenges become more complex and urgent. Many large, developed countries now have well established formalized arrangements in place for Health Technology Assessment (HTA) to inform resource allocation decision-making in healthcare. Health systems in several smaller and developing countries are also gradually turning to similar tools [2].

With per capita gross domestic product (GDP) of US$119,000 [3], a population estimated at 64,000 and high spending on health (11.6% of GDP [4]), Bermuda has achieved a relatively high average life expectancy of 81.8 [5]. Despite the high absolute and relative levels of spending on healthcare and favourable life expectancy, the health system in Bermuda faces similar challenges to other countries such as the growth and effect of non-communicable diseases (NCDs), and issues related to access to healthcare [6, 7].

The Bermuda Health Strategy 2022–2027 [8] includes an agenda to deal with the challenges of healthcare resource allocation in Bermuda along with a plan to specifically address inequality. This agenda would be well served with health outcomes measures that can be used to quantify the burden of illness, measure and track the health status of patient- and demographic- groups over time, and to perform economic evaluations of health programmes and interventions. To this end, a decision was taken to create an EQ-5D value set for Bermuda. The EQ-5D-3L and EQ-5D-5L instruments are used as health outcomes measures for reimbursement decision making in many countries.

Different countries will have different EQ-5D value sets as health-state preferences are affected by local health infrastructure, culture and other factors [9]. EQ-5D valuation studies are undertaken using protocols that ensure that the resulting index values embody certain statistical and other properties that allow them to be used as Quality Adjusted Life Year (QALY) adjustments and in other health economics applications [10]. For many years, EQ-5D has been the instrument recommended by The National Institute for Health and Care Excellence (NICE) in the United Kingdom as the measure that should be used in technology appraisal [11]. EQ-5D index values can be used to estimate the burden of an illness in QALY terms.

The 3-level instrument (EQ-5D-3L) was introduced in 1990 [12]. A 5-level version of EQ-5D has recently been developed to improve the sensitivity of the instrument- particularly for mild health-states [13]. There is a protocol for a 5-level EQ-5D valuation study [14] that requires a minimum of 1,000 respondents performing valuation tasks in face-to-face interviews with trained interviewers. In very small populations it can be extremely difficult to obtain a representative sample of 1000 respondents. In the case of Bermuda this would require a sample exceeding 3% of the adult population. In such cases, those wishing to undertake EQ-5D valuation studies may opt to begin with a 3-level valuation study to create a 3 level value set (EQ-5D-3L), based upon which a 5-level value set (EQ-5D-5L) can be developed using a crosswalk algorithm [15].

The aim of this study was to create an EQ-5D-3L value set for Bermuda, based upon which an EQ-5D-5L value set was created using a crosswalk algorithm.

## Methods

### Study design

An interviewer-administered computer-based on-line survey of a representative sample of the Bermudian population was undertaken. The elicitation process followed the English language EQ-VT version 2.1 protocol [16] adapted for the EQ-5D-3L instrument. This study received exemption from ethical approval from the University of the West Indies (Letter #CREC-SA.1307/12/2021) and ethical approval from the Ethics Committee of the Bermuda Hospitals Board (letter dated March 23rd, 2022). We have followed the CREATE checklist for reporting valuation studies [17].

### The EQ-5D-3L and EQ-5D-5L Instruments

EQ-5D is a health classification system that comprises five dimensions in order: mobility, (ability to perform) self-care, usual activities, pain/discomfort and anxiety/depression [18]. In the 3-level version of the instrument (EQ-5D-3L), a respondent can report one of three levels of problems on each dimension: no-, moderate-, or extreme- problems. The levels are coded 1, 2 and 3 respectively, allowing health states to be coded. Thus, a respondent or a patient who has no problems walking, no problems bathing or dressing themselves, some problems performing usual activities (work, study, leisure etc.), extreme pain or discomfort and moderate anxiety/depression would be in state 11232.

With 5 dimensions and 3 levels, there are 3^5^ = 243 possible states ranging from 11111 (no problems on all 5 dimensions) through 33333 (extreme problems on all dimensions). In an EQ-5D valuation study, a societal ‘value’ is obtained for each EQ-5D state. This is known as an EQ-5D index value, and it reflects the strength of preference or the value that the society places on each EQ-5D state relative to all other EQ-5D states.

The EQ VAS is another element of EQ-5D in which a respondent indicates their subjective valuation of their own state of health on a visual analogue scale ranging from 0 (worst imaginable health state) to 1 (best imaginable health state).

The EQ-5D-5L instrument was developed by including two intermediate levels: slight (between no- and moderate- problems) and severe (between moderate- and extreme- problems) on all five dimensions.

### Sample

Respondents were recruited locally in Bermuda through a marketing firm, using their respondent panel. Eligibility criteria were Bermudian citizenship and being at least 18 years old. Quota sampling was used based on age and sex with a target of 350. The recruitment effort was supported by social media awareness-building along with television and radio interviews and outreach activities in high traffic retail outlets. Respondents in Bermuda received restaurant vouchers valued at US$18.00 for their participation.

### Interview process

The interviews took place via videoconferencing software by a team of nine interviewers from a survey provider based in Trinidad and Tobago. Utilizing videoconferencing software for valuation interviews has been shown to be a viable strategy, producing results that are similar to those from conducting these interviews in a face to face setting [19, 20]. Members of the Trinidad and Tobago interviewer team had conducted two face-to-face EQ-5D valuation studies previously and had received one week of face-to-face training which was provided by two experienced EQ-5D researchers (HB and BR).

Interviewers used the EQ-VT software (version 2.1 English) for the on-line interviews. Respondents began by indicating consent and then moving to a ‘warm up task’ to build familiarity with the EQ-5D classification system by reporting their own health status on the 5 dimensions. During the valuation process, respondents were encouraged to think aloud so that interviewers could gauge their understanding of the tasks.

Respondents then moved on to the Composite Time Trade Off (cTTO) part of the survey. This began with another warm-up task to build familiarity with Time Trade Off (TTO) valuations in which respondents were asked to indicate preferences between being in a wheelchair for 10 years with being in full health for a smaller number of years. This was followed by another practice task considering either a health state much better or much worse than being in a wheelchair. Lastly, respondents practiced by valuing 3 EQ-5D-3L health states. Lead time TTO exercises were used to elicit ‘worse than dead’ valuations. This is a variant of TTO in which a lead period in full health is included in the choice task before the onset of the EQ-5D-3L state to be valued. Values for both the TTO and the lead-time TTO were subsequently inferred from the number of life years traded in those tasks (e.g. if a respondent is indifferent between 7 years in full health and 10 years in some state X, the value of that state X is 7/10 = 0.7). Data from the warm-up tasks were not used in the valuation analysis. Respondents then went on to complete 10 cTTO tasks presented in random order. Upon completion, respondents were presented the ranking of their answers to the cTTO tasks, and asked whether they agree with their answers, using the so-called feedback module.

The survey then moved on to the Discrete Choice Experiment (DCE) stage. Here respondents were presented with pairs of EQ-5D states and asked to indicate which state in each pair they preferred. One ‘warm-up’ pair was used to build familiarity with DCE tasks. Respondents then went on to complete 12 DCE tasks in random order. Details of cTTO and DCE tasks are provided elsewhere [14].

The EQ-VT protocol version 2.1 was used in the current study [16]. Although designed for the valuation of the EQ-5D-5L, similar procedures have been fielded previously for the valuation of EQ-5D-3L [21–23]. The only difference with an EQ-5D-5L valuation study using this protocol is that EQ-5D-3L health states are valued rather than EQ-5D-5L health states, and therefore the selection of states for the cTTO and DCE tasks is different from the one used in the EQ-5D-5L.

### Quality control

Quality control measures were implemented as outlined in the EQ-VT protocol [24]. The investigators monitored the data to ensure quality by checking to make sure that:

- Each interviewer took at least 3 min to explain the cTTO warm-up task
- Each interviewer explained the lead time in the worse-than-dead task
- Each respondent took at least 5 min to complete the 10 cTTO tasks
- EQ-5D state 33333 (the worst possible state) did not receive a higher value than any other state for a respondent or for an interviewer

Interviews were flagged if the interview met at least one of these criteria. If an interviewer had 4 or more flags in a batch of 10 interviews, they were retrained and the collected batch of data was removed. Furthermore, the distributions of cTTO data were compared between interviewers throughout the study, to avoid any major interviewer effects. Regular on-line quality control meetings were held to ensure interviewer compliance with these criteria during the survey period. At the start of the interviews there were quality issues with two interviewers. These interviewers received further training but their quality control reports continued to show multiple violations of the quality criteria. The two interviewers were then removed from the project and the data that they had collected were not used.

### Data analysis and modeling

The target sample size was 350 respondents to ensure that at least 100 responses were collected for each health state valued in the cTTO tasks and at least 50 responses were collected for each of the DCE choice pairs. This is similar to the sample size required in the EQ-VT protocol for EQ-5D-5L valuation studies [25] and this approach has produced good results in other EQ-5D-3L valuation studies such as those recently conducted in Russia and Tunisia [22, 23]. The same health state design was used for the cTTO elicitation in Bermuda as was used in the Russian and Tunisian studies, which includes a total of 28 health states. In the Bermuda study, each respondent valued a subset of 9 states, plus the worst health state described by the EQ-5D-3L, state 33333. For the DCE, a new health state design was generated to be used as the default design for forthcoming EQ-5D-3L valuation studies commissioned by the EuroQol Research Foundation. Priors were based on an analysis of the Russian and Tunisian EQ-5D-3L valuation studies as well as a sample of EQ-5D-3L data from a Dutch DCE survey. A Bayesian efficient design was generated, consisting of six blocks of 12 choice pairs. This mix of different priors and the Bayesian approach ensures the robustness of the design and reduces the risk of misspecification. The full design can be found in Appendix 1.

The cTTO data were analyzed using regression models correcting for heteroskedasticity. The linear regression formula takes the form of Eq. (1).

1 $$U_{j} = \beta_{0} + \beta_{1} MO2_{j} + \beta_{2} MO3_{j} + \beta_{3} SC2_{j} + \beta_{4} SC3_{j} + \beta_{5} UA2_{j} + \beta_{6} UA3_{j} + \beta_{7} PD2_{j} + \beta_{8} PD3_{j} + \beta_{9} AD2_{j} + \beta_{10} AD3_{j} + \varepsilon_{j}$$

Here, ${U}_{j}$ represents the value assigned to health state $j$. ${\beta }_{0}$ is the regression intercept, while ${\beta }_{1}$ to ${\beta }_{10}$ are the estimated weights assigned to 10 dummy variables that represent having a certain degree of problems on some dimension of the EQ-5D-3L. For example, ${PD3}_{j}$ is a dummy variable that equals 1 if the problems on pain/discomfort of state $j$ are at level 3 (extreme pain/discomfort) and 0 otherwise. Similarly, ${AD2}_{j}$ is a dummy variable that equals 1 if anxiety/depression is at level 2 in state $j$, and 0 otherwise. Together, these 10 dummy variables and their respective weights (the beta’s) can be used to assign values to all 243 possible health states.

cTTO data typically suffers from heteroskedasticity. This was corrected for by assuming that the error term ${\varepsilon }_{j}$ is normally distributed with mean 0 and variance ${\sigma }_{j}^{2}$, with ${\sigma }_{j}$ taking the form of Eq. (2): 

2 $$\sigma_{j} = {\text{exp}}\left( {\gamma_{0} + \gamma_{1} MO2_{j} + \gamma_{2} MO3_{j} + \gamma_{3} SC2_{j} + \gamma_{4} SC3_{j} + \gamma_{5} UA2_{j} + \gamma_{6} UA3_{j} + \gamma_{7} PD2_{j} + \gamma_{8} PD3_{j} + \gamma_{9} AD2_{j} + \gamma_{10} AD3_{j} } \right)$$

To model the DCE data, a full mixed logit with random parameters for each respondent was estimated, allowing for correlation between the random parameters. The utility function in the estimated model takes the form as Eq. (3) and uses a logit link function to model the binary DCE data.

3 $$U_{ij} = \beta_{i1} MO2_{ij} + \beta_{i2} MO3_{ij} + \beta_{i3} SC2_{ij} + \beta_{i4} SC3_{ij} + \beta_{i5} UA2_{ij} + \beta_{i6} UA3_{ij} + \beta_{i7} PD2_{ij} + \beta_{i8} PD3_{ij} + \beta_{i9} AD2_{ij} + \beta_{i10} AD3_{ij}$$

Finally, a hybrid model correcting for heteroskedasticity in the cTTO data was estimated. The hybrid model allows for the estimation of a joint likelihood function for the cTTO and DCE data and produces estimates on the QALY scale anchored at 0 (dead) and 1 (full health), and takes the same functional form as shown in Eq. (1).  A final model was selected based on the consistency of the parameter estimates and predictivity of the mean observed cTTO values. In case of similar performance, a hybrid model was preferred, as this allows for the utilization of all collected data.

## Results

### Sample characteristics

Data collection ran from May 2022 through June 2023. There were challenges in recruiting respondents for the survey- particularly among males in the younger age groups. Only 17.6% of the contacts received from the recruiting firm resulted in valuation interviews. A second recruiting firm was hired and the Bermuda Health Council also provided considerable support with recruitment drives through social media, television and radio interviews and distribution of flyers in high traffic retail establishments. All told 1180 prospective respondents (over 2% of the population) were contacted via e-mail directly. Of these 371 completed the EQ-VT survey. Two interviewers showed problems with protocol compliance, and in addition were not available for further interviews after conducting 5 interviews in total. This left 366 respondents for the final sample. A total of 809 respondents either refused to take part or terminated the interviews before the valuation tasks had been completed. Of the 366 interviews included in the final analyses, 68 (18.6%) were flagged. Firstly, 8.2% of interviews were flagged, as the WTD task was not entered in the wheelchair examples. Furthermore, 5.5% of interviews were flagged as they showed an inconsistency for state 33333 and 1.6% of interviews were flagged for not spending enough time on the wheelchair example. Lastly, 6.8% of interviews were flagged for not spending enough time on the cTTO tasks.

The demographics of the sample versus the population are compared in Table 1 based on the 2016 Population and Housing Census of Bermuda [26]. The sample was skewed towards older female groups with males in all age groups being under-represented.

**Table 1:** Sample Characteristics

Age Group	Sample				Population %	
	Male		Female			
	N	%	N	%	Male	Female
18–34	21	5.7%	32	8.7%	19.7%	22.0%
35–54	34	9.3%	107	29.2%	16.6%	17.7%
55 +	32	8.7%	140	38.3%	11.4%	12.6%
Total	87	23.8%	279	76.2%	47.7%	52.3%

	Sample		Population %
	N	%	
Parish			
Devonshire	41	11%	11%
Hamilton	41	11%	9%
Paget	25	7%	10%
Pembroke	37	10%	16%
Sandys	39	11%	11%
St. Georges	52	14%	9%
Smiths	31	8%	10%
Southampton	46	13%	10%
Warwick	54	15%	14%

Ethnicity			
Black	221	60%	52%
White	107	29%	31%
Other	38	10%	17%

Just over half (50.8%) of the respondents reported being in state 11111 with the most common level 2 dimensions being pain/discomfort (34.7%) and mobility (14.5%). The mean EQ VAS score was 80.4 (standard deviation = 13.7).

### Data analysis and modeling

3660 cTTO responses were collected, of which 764 were worse than dead (20.9%). The mean observed values for each health state are reported in Table 2.

**Table 2:** Observed TTO values

State	Mean	SE
11112	0.859	0.026
11121	0.843	0.026
11122	0.746	0.035
11211	0.882	0.029
11313	0.454	0.044
12111	0.862	0.031
12212	0.684	0.032
12331	0.216	0.050
13133	0.038	0.056
13221	0.559	0.037
21111	0.881	0.024
21133	0.167	0.056
21211	0.850	0.027
21323	0.387	0.050
21332	0.135	0.051
22121	0.727	0.033
22222	0.518	0.041
22233	0.067	0.062
23112	0.518	0.042
23323	0.096	0.057
31131	0.144	0.054
31223	0.007	0.056
32113	0.163	0.056
32232	– 0.168	0.056
32322	0.147	0.053
33232	– 0.103	0.059
33311	0.144	0.058
33333	– 0.392	0.033

Only analytical approaches that corrected for the representativeness issue were considered. First, a weighted regression model corrected for heteroskedasticity on the cTTO data and a weighted mixed logit regression analysis on the DCE data both provided preliminary models that had internally consistent, significant (p < 0.05) coefficients. Both of these weighted regression models used inverse sampling weights to allocate a larger weight to respondents from age-gender groups that were underrepresented and smaller weights to groups that were overrepresented. These preliminary models are displayed in Table 3.

**Table 3:** Preliminary models

	TTO Heteroskedastic		Mixed Logit		
	Coefficient	SE	Coefficient	SD	Rescaled
MO2	0.101	0.017	0.932	0.169	0.061
MO3	0.402	0.029	6.075	0.674	0.396
SC2	0.110	0.022	1.269	0.169	0.083
SC3	0.234	0.027	3.331	0.389	0.217
UA2	0.093	0.021	0.882	0.164	0.057
UA3	0.143	0.026	2.752	0.364	0.179
PD2	0.119	0.019	1.800	0.222	0.117
PD3	0.407	0.027	6.402	0.737	0.417
AD2	0.125	0.019	1.448	0.188	0.094
AD3	0.294	0.026	4.171	0.497	0.272
AIC	5041.677		5217.938		
BIC	5171.987		5678.183		

*AIC* akaike information criterion, *BIC* Bayesian information criterion

A hybrid model correcting for heteroskedasticity was estimated to capture the advantages of both the TTO and DCE valuation approaches. However, hybrid models do not allow for sampling weight corrections in the software packages in which they are available (Stata and R). Therefore, a bootstrap procedure was used in which random draws of subsamples of the overrepresented sample groups were drawn to produce smaller representative samples upon which the hybrid models were subsequently estimated. The largest subsample of the full sample that was representative of the Bermudan population included n = 151 respondents. This includes the full sample of underrepresented quota groups given this sample size, and a proportional random draw of each the other quota groups. A total of 3000 of such draws were done, and for each of these draws a hybrid model was estimated. A distribution of the modelled coefficients is reported in Table 4. Given that most of the EQ-5D-3L value sets currently in use are TTO based [27], a similar procedure was conducted on the TTO data in order to investigate the level of similarity between the TTO model and the hybrid model. The two bootstrapped models are presented in Table 4. The bootstrapped models showed similar fit to the mean observed cTTO responses for the 28 states included in the cTTO task (mean absolute deviation vs observed cTTO responses was 0.05 for both the hybrid model and the cTTO model). Sensitivity analyses were conducted by excluding feedback module flagged states from the sample (347 responses or 6.3%) or by preserving the 5 interviews that were removed from analysis, but neither had any effect on the estimated values. Finally, the hybrid model was selected to generate the EQ-5D-3L value set for Bermuda, as while the models showed similar fit and estimates, the hybrid model allows for the use of all collected data, rather than just the cTTO data.

**Table 4:** Heteroskedastic TTO and Hybrid models: bootstrap analysis with 3000 random samples

	Hybrid Model		TTO model for Comparison	
	Beta	SD	Beta	SD
MO2	0.061	0.005	0.091	0.012
MO3	0.427	0.013	0.394	0.020
SC2	0.097	0.006	0.111	0.012
SC3	0.225	0.008	0.229	0.013
UA2	0.065	0.004	0.085	0.011
UA3	0.186	0.007	0.166	0.016
PD2	0.126	0.006	0.121	0.009
PD3	0.421	0.013	0.407	0.015
AD2	0.110	0.006	0.123	0.015
AD3	0.288	0.009	0.298	0.017

The value of any state can easily be calculated by subtracting these beta values from the value of the full health state (11111) which is set at 1.000 by definition. For example, using the hybrid model in Table 4, the value associated with state 21322 would be 1.000 – 0.061– 0.000 – 0.186 – 0.126 – 0.110 = 0.517. The zero is included in this calculation for level 1 on self-care in state 21322, as there is no reduction in index value for a dimension at level 1 (no problems). The coefficients from the two models in Table 4 are very similar. A mean absolute deviation of 0.027 was observed between the value sets generated by these two models.

At level 2 (moderate problems), pain/discomfort and anxiety/depression have the biggest effect on index values. At level 3 (extreme problems), mobility and pain/discomfort have the biggest effect. Out of the 243 EQ-5D-3L states, 34 had negative values with the lowest valued state being 33333 with a value of – 0.547.

**Fig. 1:**
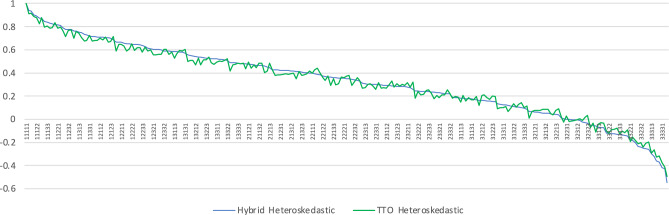
Similarity between the value sets generated by the Hybrid and TTO Bootstrap Models

**Fig. 2:**
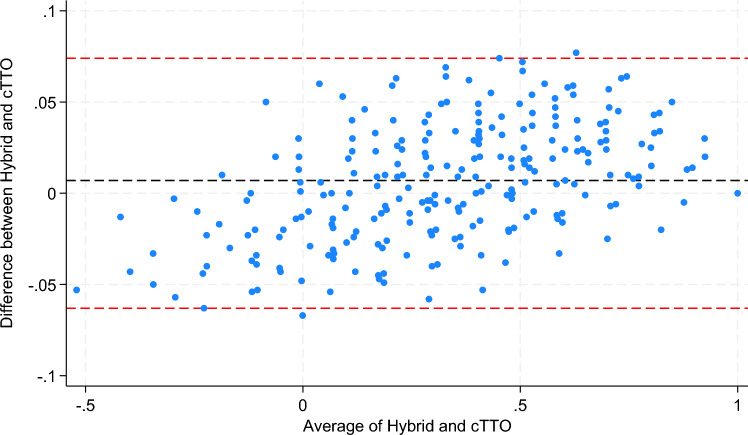
Bland–Altman Plot for the value sets generated by the Hybrid and TTO Bootstrap Models

Figure 1 shows the closeness of the values from the two models across the value set, and the Bland Altman plot in Fig. 2 shows that fewer than 3% of the states have differences outside of the 95% confidence interval between the two models.

The EQ-5D-3L value set and the EQ-5D-5L Crosswalk set are presented in Appendices 2 and 3.

## Conclusion

This study has produced an EQ-5D-3L value set for Bermuda. The hybrid model produced values that were very similar to those obtained from the cTTO data only.

Table 5 shows a comparison of the Bermuda EQ-5D-3L coefficients with those of the three other countries for which EQ-5D-3L valuation studies have been undertaken using EQ-VT: Russia, Pakistan and Tunisia. Also included in Table 5 are another island state: Trinidad and Tobago along with the countries listed in an inventory of TTO-based valuation studies [27]. While the coefficients may not be directly comparable because of the inclusion of interaction (and other) terms, along with differences in valuation protocols (as not all of the other countries in Table 5 utilized EQ-VT) some differences can be discerned between preferences in Bermuda and those in other countries. The Bermuda value set differs from the DCE-TTO based 3-level value set from Trinidad and Tobago: there were fewer negative values and the highest impact dimensions were pain/discomfort and mobility at levels 2 and 3 [28]. The Bermuda 3-level value set also differs from those of other nearby countries: the highest level 2 coefficients were pain and self-care in the United Kingdom and the United States, while for level 3 pain/discomfort had the biggest coefficient for both countries but self-care was second highest for the United States and mobility was second highest for the United Kingdom [27]. Such differences highlight the need for using local values in resource allocation and policy making in healthcare [9].

**Table 5:** Bermuda EQ-5D-3L coefficients compared with those of other countries

	Bermuda	Russia	Pakistan	Tunisia	Trinidad and Tobago	USA	UK	Netherlands	Spain	Denmark	Japan	Zimbabwe
Constant	–	–	– 0.030		– 0.093	–	– 0.081	– 0.071	– 0.024	– 0.114	– 0.152	– 0.100
MO2	– 0.061	– 0.049	– 0.034	– 0.076	– 0.045	– 0.146	– 0.069	– 0.036	– 0.106	– 0.053	– 0.075	– 0.056
MO3	– 0.427	– 0.448	– 0.323	– 0.597	– 0.412	– 0.558	– 0.314	– 0.161	– 0.430	– 0.411	– 0.418	– 0.204
SC2	– 0.097	– 0.081	– 0.060	– 0.165	– 0.064	– 0.175	– 0.104	– 0.082	– 0.134	– 0.063	– 0.054	– 0.092
SC3	– 0.225	– 0.230	– 0.275	– 0.340	– 0.172	– 0.471	– 0.214	– 0.152	– 0.309	– 0.192	– 0.102	– 0.231
UA2	– 0.065	– 0.075	– 0.041	– 0.078	– 0.043	– 0.140	– 0.036	– 0.032	– 0.071	– 0.048	– 0.044	– 0.043
UA3	– 0.186	– 0.236	– 0.352	– 0.251	– 0.117	– 0.374	– 0.094	– 0.057	– 0.195	– 0.144	– 0.133	– 0.135
PD2	– 0.126	– 0.069	– 0.018	– 0.057	– 0.064	– 0.173	– 0.123	– 0.086	– 0.089	– 0.062	– 0.080	– 0.067
PD3	– 0.421	– 0.369	– 0.255	– 0.276	– 0.230	– 0.537	– 0.386	– 0.329	– 0.261	– 0.396	– 0.194	– 0.302
AD2	– 0.110	– 0.028	– 0.025	– 0.095	– 0.011	– 0.156	– 0.071	– 0.124	– 0.062	– 0.068	– 0.063	– 0.046
AD3	– 0.288	– 0.172	– 0.218	– 0.332	– 0.139	– 0.450	– 0.236	– 0.325	– 0.144	– 0.367	– 0.112	– 0.173
N3	–	–	–	–	–	–	– 0.269	– 0.234	– 0.291	–	–	–
D1	–	–	–	–	–	0.14	–	–	–	–	–	–
I2-Square	–	–	–	–	–	– 0.011	–	–	–	–	–	–
I3	–	–	–	–	–	0.122	–	–	–	–	–	–
I3-Square	–	–	–	–	–	0.015	–	–	–	–	–	–

D1: The number of movements away from state 11,111 beyond the first one, I3: The number of dimensions at level 3 beyond the first one, I2: The number of dimensions at level 2 beyond the first one, N3: At least one dimension at level 3

There were some challenges with sampling; it was difficult to collect a representative sample of around 350 individuals in a country with a population of 64,000. This led to an overrepresentation of females in the older age categories. A viable analytical solution had to be found to account for the over-sampling of some groups, and the under-sampling of others. Weighted models are usually the preferred method, but these rely on sufficient data within each quota group; a criterion that was not met for some of the age-sex groups. To deal with this, some quota groups were collapsed. As weighted models are not supported for hybrid models, a bootstrap approach was used in this study which provides a robust estimate of what would be expected from a single model estimated on a representative sample. Further studies can be undertaken to test the level of similarity of the preferences in the underrepresented age-gender groups to those in Table 4. Furthermore, the high level of agreement observed between the weighted model on the TTO data and the bootstrap of the TTO data suggest that the bootstrap approach is a viable alternative to using a weighted model for the hybrid model.

The idea of conducting EQ-5D valuation studies on-line with an experienced Trinidad-based team using the EQ-VT platform opens the possibility of doing similar studies in the smaller Caribbean and Central American countries. The quality control protocol of EQ-VT in Sect. 2.5, the resulting feedback and actions discussed in the "Sample Characteristics" section, and the performance of the final model in the "Data Analysis and Modeling" section all point to this modality being useful/appropriate for the subregion. This can greatly reduce the cost of conducting such studies locally. The alternative to this would be to train and supervise teams of interviewers locally for the valuation study in each country. One learning that came out of the Bermuda valuation study was associated with the possible challenge of recruiting respondents for an on-line survey. Especially when working with small populations, careful consideration should be given to the possibility of high refusal rates which may in part be associated with outreach from overseas. Strong local promotion and recruitment efforts before initializing outreach from the valuation team should help in such circumstances.

The values created in this study can be used in the economic analysis of health interventions and to assess the burden of disease of different patient groups and demographic groups in Bermuda. As Bermuda moves towards developing its own HTA arrangements, applying societal values to self-reported health states can provide unique insights into understanding the burden of disease and can capture aspects of health inequality that can be missed by other measures. Self-reported health-based measures bring the patients’ or respondents’ experience with their health states directly into the analysis. Such studies continue to bring new insights into burden of disease and health inequality in the small islands of the Caribbean and other countries/regions [29, 30].

## Appendix 1. DCE design

**Table:**

BLOCK_ID	STATE_ID	MO_A	SC_A	UA_A	PD_A	AD_A	MO_B	SC_B	UA_B	PD_B	AD_B
**1**	1	2	2	2	1	1	1	3	1	1	1
1	2	1	2	2	2	2	2	1	1	2	2
1	3	3	3	1	3	2	3	3	3	2	3
1	4	1	2	3	2	2	1	1	3	3	1
1	5	3	1	3	2	1	3	1	2	1	3
1	6	1	1	3	3	3	2	3	1	3	3
1	7	2	3	2	2	1	2	3	3	1	2
1	8	3	2	1	1	1	3	1	1	2	2
1	9	2	2	2	3	3	3	1	2	1	3
1	10	1	2	1	3	3	1	3	2	3	2
1	11	2	2	3	1	3	3	2	1	2	3
1	12	3	2	3	1	2	1	3	3	1	3
2	13	2	1	2	3	2	2	3	2	2	1
2	14	1	3	1	2	1	2	2	1	1	1
2	15	1	2	2	3	1	2	1	1	3	1
2	16	3	2	1	3	1	3	1	2	3	2
2	17	3	3	2	3	1	3	3	3	1	3
2	18	1	1	3	2	3	2	3	3	2	2
2	19	2	1	2	2	3	3	1	2	1	1
2	20	1	2	3	1	2	2	2	2	1	3
2	21	1	1	3	2	1	1	2	1	2	2
2	22	3	1	1	2	3	1	3	1	3	3
2	23	3	1	1	3	2	2	2	3	3	2
2	24	3	3	2	1	2	3	2	3	2	2
3	25	1	2	2	1	2	1	1	1	1	3
3	26	2	2	2	3	3	2	3	3	3	1
3	27	3	1	3	2	1	2	3	3	3	1
3	28	2	3	1	2	1	1	3	1	1	3
3	29	1	1	3	1	2	1	3	2	1	1
3	30	3	2	3	3	1	3	3	1	3	2
3	31	2	2	1	1	2	2	1	2	2	2
3	32	1	3	2	2	3	2	1	2	3	3
3	33	1	1	1	3	1	2	1	1	1	3
3	34	3	1	2	1	1	3	2	1	2	1
3	35	3	2	2	2	3	1	2	1	3	3
3	36	2	3	3	2	2	3	3	1	1	2
4	37	1	1	3	1	1	2	2	2	1	1
4	38	1	3	1	1	3	1	3	2	2	2
4	39	3	3	3	2	3	3	2	3	3	1
4	40	2	1	3	1	2	1	2	3	2	2
4	41	1	2	3	3	3	3	1	1	3	3
4	42	2	1	2	2	3	2	3	1	2	2
4	43	2	2	3	3	1	1	3	2	3	1
4	44	3	3	3	1	1	3	2	1	1	3
4	45	1	1	2	1	2	1	1	1	2	1
4	46	2	3	2	3	2	3	2	2	1	2
4	47	2	1	3	2	2	1	1	3	3	1
4	48	2	3	1	1	1	2	1	1	3	2
5	49	1	2	2	2	2	2	2	1	2	1
5	50	1	2	3	1	1	2	1	3	1	2
5	51	3	2	3	1	3	3	3	1	2	3
5	52	2	2	2	3	2	1	1	2	3	3
5	53	3	3	2	2	2	3	3	1	1	3
5	54	3	1	3	3	1	2	3	3	3	3
5	55	1	2	2	2	3	1	2	1	3	2
5	56	3	3	2	3	1	3	3	3	1	2
5	57	2	1	2	3	3	3	1	1	2	3
5	58	2	2	1	2	3	2	3	3	2	1
5	59	3	1	2	1	1	1	1	3	2	1
5	60	1	2	3	2	3	1	3	2	1	3
6	61	3	1	1	3	2	1	2	3	3	2
6	62	1	3	2	1	3	2	3	3	1	2
6	63	3	2	3	2	3	3	2	2	3	1
6	64	2	3	1	2	1	2	1	3	1	1
6	65	1	3	2	2	1	1	1	1	2	3
6	66	1	3	3	3	2	2	3	2	3	3
6	67	2	2	1	1	2	2	1	1	2	1
6	68	2	3	3	3	3	3	2	3	2	3
6	69	1	1	1	2	2	1	1	3	1	1
6	70	3	2	1	3	1	3	1	2	3	2
6	71	1	3	2	1	1	2	2	2	2	1
6	72	2	3	1	1	2	1	2	1	3	2

## Appendix 2. The EQ-5D-3L value set for Bermuda

**Table:**

State	Value	State	Value	State	Value	State	Value
11111	1.000	13132	0.244	22223	0.363	31321	0.261
11112	0.890	13133	0.066	22231	0.356	31322	0.151
11113	0.712	13211	0.710	22232	0.246	31323	– 0.027
11121	0.874	13212	0.600	22233	0.068	31331	– 0.034
11122	0.764	13213	0.422	22311	0.656	31332	– 0.144
11123	0.586	13221	0.584	22312	0.546	31333	– 0.322
11131	0.579	13222	0.474	22313	0.368	32111	0.476
11132	0.469	13223	0.296	22321	0.530	32112	0.366
11133	0.291	13231	0.289	22322	0.420	32113	0.188
11211	0.935	13232	0.179	22323	0.242	32121	0.350
11212	0.825	13233	0.001	22331	0.235	32122	0.240
11213	0.647	13311	0.589	22332	0.125	32123	0.062
11221	0.809	13312	0.479	22333	– 0.053	32131	0.055
11222	0.699	13313	0.301	23111	0.714	32132	– 0.055
11223	0.521	13321	0.463	23112	0.604	32133	– 0.233
11231	0.514	13322	0.353	23113	0.426	32211	0.411
11232	0.404	13323	0.175	23121	0.588	32212	0.301
11233	0.226	13331	0.168	23122	0.478	32213	0.123
11311	0.814	13332	0.058	23123	0.300	32221	0.285
11312	0.704	13333	– 0.120	23131	0.293	32222	0.175
11313	0.526	21111	0.939	23132	0.183	32223	– 0.003
11321	0.688	21112	0.829	23133	0.005	32231	– 0.010
11322	0.578	21113	0.651	23211	0.649	32232	– 0.120
11323	0.400	21121	0.813	23212	0.539	32233	– 0.298
11331	0.393	21122	0.703	23213	0.361	32311	0.290
11332	0.283	21123	0.525	23221	0.523	32312	0.180
11333	0.105	21131	0.518	23222	0.413	32313	0.002
12111	0.903	21132	0.408	23223	0.235	32321	0.164
12112	0.793	21133	0.230	23231	0.228	32322	0.054
12113	0.615	21211	0.874	23232	0.118	32323	– 0.124
12121	0.777	21212	0.764	23233	– 0.060	32331	– 0.131
12122	0.667	21213	0.586	23311	0.528	32332	– 0.241
12123	0.489	21221	0.748	23312	0.418	32333	– 0.419
12131	0.482	21222	0.638	23313	0.240	33111	0.348
12132	0.372	21223	0.460	23321	0.402	33112	0.238
12133	0.194	21231	0.453	23322	0.292	33113	0.060
12211	0.838	21232	0.343	23323	0.114	33121	0.222
12212	0.728	21233	0.165	23331	0.107	33122	0.112
12213	0.550	21311	0.753	23332	– 0.003	33123	– 0.066
12221	0.712	21312	0.643	23333	– 0.181	33131	– 0.073
12222	0.602	21313	0.465	31111	0.573	33132	– 0.183
12223	0.424	21321	0.627	31112	0.463	33133	– 0.361
12231	0.417	21322	0.517	31113	0.285	33211	0.283
12232	0.307	21323	0.339	31121	0.447	33212	0.173
12233	0.129	21331	0.332	31122	0.337	33213	– 0.005
12311	0.717	21332	0.222	31123	0.159	33221	0.157
12312	0.607	21333	0.044	31131	0.152	33222	0.047
12313	0.429	22111	0.842	31132	0.042	33223	– 0.131
12321	0.591	22112	0.732	31133	– 0.136	33231	– 0.138
12322	0.481	22113	0.554	31211	0.508	33232	– 0.248
12323	0.303	22121	0.716	31212	0.398	33233	– 0.426
12331	0.296	22122	0.606	31213	0.220	33311	0.162
12332	0.186	22123	0.428	31221	0.382	33312	0.052
12333	0.008	22131	0.421	31222	0.272	33313	– 0.126
13111	0.775	22132	0.311	31223	0.094	33321	0.036
13112	0.665	22133	0.133	31231	0.087	33322	– 0.074
13113	0.487	22211	0.777	31232	– 0.023	33323	– 0.252
13121	0.649	22212	0.667	31233	– 0.201	33331	– 0.259
13122	0.539	22213	0.489	31311	0.387	33332	– 0.369
13123	0.361	22221	0.651	31312	0.277	33333	– 0.547
13131	0.354	22222	0.541	31313	0.099		

## Appendix 3. The EQ-5D-5L crosswalk set for Bermuda

**Table:**

State	Value	State	Value	State	Value	State	Value	State	Value	State	Value	State	Value
11111	1.000	11331	0.809	11551	0.393	12321	0.753	12541	0.489	13311	0.838	13531	0.591
11112	0.913	11332	0.722	11552	0.306	12322	0.666	12542	0.402	13312	0.751	13532	0.504
11113	0.890	11333	0.699	11553	0.283	12323	0.643	12543	0.379	13313	0.728	13533	0.481
11114	0.803	11334	0.612	11554	0.196	12324	0.556	12544	0.292	13314	0.641	13534	0.394
11115	0.712	11335	0.521	11555	0.105	12325	0.465	12545	0.201	13315	0.550	13535	0.303
11121	0.899	11341	0.691	12111	0.919	12331	0.728	12551	0.312	13321	0.737	13541	0.473
11122	0.812	11342	0.604	12112	0.832	12332	0.641	12552	0.225	13322	0.650	13542	0.386
11123	0.789	11343	0.581	12113	0.809	12333	0.618	12553	0.202	13323	0.627	13543	0.363
11124	0.702	11344	0.494	12114	0.722	12334	0.531	12554	0.115	13324	0.540	13544	0.276
11125	0.611	11345	0.403	12115	0.631	12335	0.440	12555	0.024	13325	0.449	13545	0.185
11131	0.874	11351	0.514	12121	0.818	12341	0.610	13111	0.903	13331	0.712	13551	0.296
11132	0.787	11352	0.427	12122	0.731	12342	0.523	13112	0.816	13332	0.625	13552	0.209
11133	0.764	11353	0.404	12123	0.708	12343	0.500	13113	0.793	13333	0.602	13553	0.186
11134	0.677	11354	0.317	12124	0.621	12344	0.413	13114	0.706	13334	0.515	13554	0.099
11135	0.586	11355	0.226	12125	0.530	12345	0.322	13115	0.615	13335	0.424	13555	0.008
11141	0.756	11411	0.895	12131	0.793	12351	0.433	13121	0.802	13341	0.594	14111	0.872
11142	0.669	11412	0.808	12132	0.706	12352	0.346	13122	0.715	13342	0.507	14112	0.785
11143	0.646	11413	0.785	12133	0.683	12353	0.323	13123	0.692	13343	0.484	14113	0.762
11144	0.559	11414	0.698	12134	0.596	12354	0.236	13124	0.605	13344	0.397	14114	0.675
11145	0.468	11415	0.607	12135	0.505	12355	0.145	13125	0.514	13345	0.306	14115	0.584
11151	0.579	11421	0.794	12141	0.675	12411	0.814	13131	0.777	13351	0.417	14121	0.771
11152	0.492	11422	0.707	12142	0.588	12412	0.727	13132	0.690	13352	0.330	14122	0.684
11153	0.469	11423	0.684	12143	0.565	12413	0.704	13133	0.667	13353	0.307	14123	0.661
11154	0.382	11424	0.597	12144	0.478	12414	0.617	13134	0.580	13354	0.220	14124	0.574
11155	0.291	11425	0.506	12145	0.387	12415	0.526	13135	0.489	13355	0.129	14125	0.483
11211	0.948	11431	0.769	12151	0.498	12421	0.714	13141	0.659	13411	0.798	14131	0.746
11212	0.861	11432	0.682	12152	0.411	12422	0.626	13142	0.572	13412	0.711	14132	0.659
11213	0.838	11433	0.659	12153	0.388	12423	0.604	13143	0.549	13413	0.688	14133	0.636
11214	0.751	11434	0.572	12154	0.301	12424	0.516	13144	0.462	13414	0.601	14134	0.549
11215	0.660	11435	0.481	12155	0.210	12425	0.426	13145	0.371	13415	0.510	14135	0.458
11221	0.847	11441	0.651	12211	0.867	12431	0.688	13151	0.482	13421	0.697	14141	0.628
11222	0.760	11442	0.564	12212	0.780	12432	0.601	13152	0.395	13422	0.610	14142	0.541
11223	0.737	11443	0.541	12213	0.757	12433	0.578	13153	0.372	13423	0.587	14143	0.518
11224	0.650	11444	0.454	12214	0.670	12434	0.491	13154	0.285	13424	0.500	14144	0.431
11225	0.559	11445	0.363	12215	0.579	12435	0.400	13155	0.194	13425	0.409	14145	0.340
11231	0.822	11451	0.474	12221	0.766	12441	0.571	13211	0.851	13431	0.672	14151	0.451
11232	0.735	11452	0.387	12222	0.679	12442	0.483	13212	0.764	13432	0.585	14152	0.364
11233	0.712	11453	0.364	12223	0.656	12443	0.461	13213	0.741	13433	0.562	14153	0.341
11234	0.625	11454	0.277	12224	0.569	12444	0.373	13214	0.654	13434	0.475	14154	0.254
11235	0.534	11455	0.186	12225	0.478	12445	0.283	13215	0.563	13435	0.384	14155	0.163
11241	0.704	11511	0.814	12231	0.741	12451	0.393	13221	0.750	13441	0.554	14211	0.820
11242	0.617	11512	0.727	12232	0.654	12452	0.306	13222	0.663	13442	0.467	14212	0.732
11243	0.594	11513	0.704	12233	0.631	12453	0.283	13223	0.640	13443	0.444	14213	0.710
11244	0.507	11514	0.617	12234	0.544	12454	0.196	13224	0.553	13444	0.357	14214	0.622
11245	0.416	11515	0.526	12235	0.453	12455	0.105	13225	0.462	13445	0.266	14215	0.532
11251	0.527	11521	0.713	12241	0.623	12511	0.733	13231	0.725	13451	0.377	14221	0.719
11252	0.440	11522	0.626	12242	0.536	12512	0.646	13232	0.638	13452	0.290	14222	0.632
11253	0.417	11523	0.603	12243	0.513	12513	0.623	13233	0.615	13453	0.267	14223	0.609
11254	0.330	11524	0.516	12244	0.426	12514	0.536	13234	0.528	13454	0.180	14224	0.521
11255	0.239	11525	0.425	12245	0.335	12515	0.445	13235	0.437	13455	0.089	14225	0.431
11311	0.935	11531	0.688	12251	0.446	12521	0.632	13241	0.607	13511	0.717	14231	0.694
11312	0.848	11532	0.601	12252	0.359	12522	0.545	13242	0.520	13512	0.630	14232	0.606
11313	0.825	11533	0.578	12253	0.336	12523	0.522	13243	0.497	13513	0.607	14233	0.584
11314	0.738	11534	0.491	12254	0.249	12524	0.435	13244	0.410	13514	0.520	14234	0.496
11315	0.647	11535	0.400	12255	0.158	12525	0.344	13245	0.319	13515	0.429	14235	0.406
11321	0.834	11541	0.570	12311	0.854	12531	0.607	13251	0.430	13521	0.616	14241	0.576
11322	0.747	11542	0.483	12312	0.767	12532	0.520	13252	0.343	13522	0.529	14242	0.489
11323	0.724	11543	0.460	12313	0.744	12533	0.497	13253	0.320	13523	0.506	14243	0.466
11324	0.637	11544	0.373	12314	0.657	12534	0.410	13254	0.233	13524	0.419	14244	0.379
11325	0.546	11545	0.282	12315	0.566	12535	0.319	13255	0.142	13525	0.328	14245	0.288
14251	0.399	14521	0.585	15241	0.479	15511	0.589	21231	0.772	21451	0.424	22221	0.716
14252	0.311	14522	0.498	15242	0.392	15512	0.502	21232	0.684	21452	0.337	22222	0.629
14253	0.289	14523	0.475	15243	0.369	15513	0.479	21233	0.662	21453	0.314	22223	0.606
14254	0.201	14524	0.388	15244	0.282	15514	0.392	21234	0.574	21454	0.227	22224	0.519
14255	0.111	14525	0.297	15245	0.191	15515	0.301	21235	0.484	21455	0.136	22225	0.428
14311	0.807	14531	0.560	15251	0.302	15521	0.488	21241	0.654	21511	0.764	22231	0.691
14312	0.720	14532	0.473	15252	0.215	15522	0.401	21242	0.567	21512	0.677	22232	0.604
14313	0.697	14533	0.450	15253	0.192	15523	0.378	21243	0.544	21513	0.654	22233	0.581
14314	0.610	14534	0.363	15254	0.105	15524	0.291	21244	0.456	21514	0.566	22234	0.494
14315	0.519	14535	0.272	15255	0.014	15525	0.200	21245	0.366	21515	0.476	22235	0.403
14321	0.706	14541	0.442	15311	0.710	15531	0.463	21251	0.477	21521	0.663	22241	0.573
14322	0.619	14542	0.355	15312	0.623	15532	0.376	21252	0.389	21522	0.576	22242	0.486
14323	0.596	14543	0.332	15313	0.600	15533	0.353	21253	0.367	21523	0.553	22243	0.463
14324	0.509	14544	0.245	15314	0.513	15534	0.266	21254	0.279	21524	0.466	22244	0.376
14325	0.418	14545	0.154	15315	0.422	15535	0.175	21255	0.189	21525	0.375	22245	0.285
14331	0.681	14551	0.265	15321	0.609	15541	0.345	21311	0.885	21531	0.638	22251	0.396
14332	0.594	14552	0.178	15322	0.522	15542	0.258	21312	0.798	21532	0.551	22252	0.309
14333	0.571	14553	0.155	15323	0.499	15543	0.235	21313	0.775	21533	0.528	22253	0.286
14334	0.484	14554	0.068	15324	0.412	15544	0.148	21314	0.687	21534	0.440	22254	0.199
14335	0.393	14555	– 0.023	15325	0.321	15545	0.057	21315	0.597	21535	0.350	22255	0.108
14341	0.563	15111	0.775	15331	0.584	15551	0.168	21321	0.784	21541	0.520	22311	0.804
14342	0.476	15112	0.688	15332	0.497	15552	0.081	21322	0.697	21542	0.433	22312	0.717
14343	0.453	15113	0.665	15333	0.474	15553	0.058	21323	0.674	21543	0.410	22313	0.694
14344	0.366	15114	0.578	15334	0.387	15554	– 0.029	21324	0.587	21544	0.323	22314	0.607
14345	0.275	15115	0.487	15335	0.296	15555	– 0.120	21325	0.496	21545	0.232	22315	0.516
14351	0.386	15121	0.674	15341	0.466	21111	0.950	21331	0.759	21551	0.343	22321	0.703
14352	0.299	15122	0.587	15342	0.379	21112	0.863	21332	0.672	21552	0.256	22322	0.616
14353	0.276	15123	0.564	15343	0.356	21113	0.840	21333	0.649	21553	0.233	22323	0.593
14354	0.189	15124	0.477	15344	0.269	21114	0.752	21334	0.561	21554	0.145	22324	0.506
14355	0.098	15125	0.386	15345	0.178	21115	0.662	21335	0.471	21555	0.055	22325	0.415
14411	0.767	15131	0.649	15351	0.289	21121	0.849	21341	0.641	22111	0.869	22331	0.678
14412	0.680	15132	0.562	15352	0.202	21122	0.762	21342	0.554	22112	0.782	22332	0.591
14413	0.657	15133	0.539	15353	0.179	21123	0.739	21343	0.531	22113	0.759	22333	0.568
14414	0.570	15134	0.452	15354	0.092	21124	0.652	21344	0.444	22114	0.672	22334	0.481
14415	0.479	15135	0.361	15355	0.001	21125	0.561	21345	0.353	22115	0.581	22335	0.390
14421	0.666	15141	0.531	15411	0.670	21131	0.824	21351	0.464	22121	0.768	22341	0.560
14422	0.579	15142	0.444	15412	0.583	21132	0.737	21352	0.377	22122	0.681	22342	0.473
14423	0.556	15143	0.421	15413	0.560	21133	0.714	21353	0.354	22123	0.658	22343	0.450
14424	0.469	15144	0.334	15414	0.473	21134	0.626	21354	0.266	22124	0.571	22344	0.363
14425	0.378	15145	0.243	15415	0.382	21135	0.536	21355	0.176	22125	0.480	22345	0.272
14431	0.641	15151	0.354	15421	0.569	21141	0.706	21411	0.845	22131	0.743	22351	0.383
14432	0.554	15152	0.267	15422	0.482	21142	0.619	21412	0.758	22132	0.656	22352	0.296
14433	0.531	15153	0.244	15423	0.459	21143	0.596	21413	0.735	22133	0.633	22353	0.273
14434	0.444	15154	0.157	15424	0.372	21144	0.509	21414	0.648	22134	0.546	22354	0.186
14435	0.353	15155	0.066	15425	0.281	21145	0.418	21415	0.557	22135	0.455	22355	0.095
14441	0.523	15211	0.723	15431	0.544	21151	0.529	21421	0.744	22141	0.625	22411	0.764
14442	0.436	15212	0.636	15432	0.457	21152	0.442	21422	0.657	22142	0.538	22412	0.677
14443	0.413	15213	0.613	15433	0.434	21153	0.419	21423	0.634	22143	0.515	22413	0.654
14444	0.326	15214	0.526	15434	0.347	21154	0.331	21424	0.547	22144	0.428	22414	0.567
14445	0.235	15215	0.435	15435	0.256	21155	0.241	21425	0.456	22145	0.337	22415	0.476
14451	0.346	15221	0.622	15441	0.426	21211	0.898	21431	0.719	22151	0.448	22421	0.663
14452	0.259	15222	0.535	15442	0.339	21212	0.810	21432	0.632	22152	0.361	22422	0.576
14453	0.236	15223	0.512	15443	0.316	21213	0.788	21433	0.609	22153	0.338	22423	0.553
14454	0.149	15224	0.425	15444	0.229	21214	0.700	21434	0.522	22154	0.251	22424	0.466
14455	0.058	15225	0.334	15445	0.138	21215	0.610	21435	0.431	22155	0.160	22425	0.375
14511	0.686	15231	0.597	15451	0.249	21221	0.797	21441	0.601	22211	0.817	22431	0.638
14512	0.599	15232	0.510	15452	0.162	21222	0.709	21442	0.514	22212	0.730	22432	0.551
14513	0.576	15233	0.487	15453	0.139	21223	0.687	21443	0.491	22213	0.707	22433	0.528
14514	0.489	15234	0.400	15454	0.052	21224	0.599	21444	0.404	22214	0.620	22434	0.441
14515	0.398	15235	0.309	15455	– 0.039	21225	0.509	21445	0.313	22215	0.529	22435	0.350
22441	0.520	23211	0.801	23431	0.622	24151	0.401	24421	0.616	25141	0.481	25411	0.620
22442	0.433	23212	0.713	23432	0.535	24152	0.313	24422	0.529	25142	0.394	25412	0.533
22443	0.410	23213	0.691	23433	0.512	24153	0.291	24423	0.506	25143	0.371	25413	0.510
22444	0.323	23214	0.603	23434	0.425	24154	0.203	24424	0.419	25144	0.284	25414	0.423
22445	0.232	23215	0.513	23435	0.334	24155	0.113	24425	0.328	25145	0.193	25415	0.332
22451	0.343	23221	0.700	23441	0.504	24211	0.770	24431	0.591	25151	0.304	25421	0.519
22452	0.256	23222	0.612	23442	0.417	24212	0.682	24432	0.504	25152	0.217	25422	0.432
22453	0.233	23223	0.590	23443	0.394	24213	0.660	24433	0.481	25153	0.194	25423	0.409
22454	0.146	23224	0.502	23444	0.307	24214	0.572	24434	0.394	25154	0.106	25424	0.322
22455	0.055	23225	0.412	23445	0.216	24215	0.482	24435	0.303	25155	0.016	25425	0.231
22511	0.683	23231	0.675	23451	0.327	24221	0.669	24441	0.473	25211	0.673	25431	0.494
22512	0.596	23232	0.587	23452	0.240	24222	0.581	24442	0.386	25212	0.585	25432	0.407
22513	0.573	23233	0.565	23453	0.217	24223	0.559	24443	0.363	25213	0.563	25433	0.384
22514	0.486	23234	0.477	23454	0.130	24224	0.471	24444	0.276	25214	0.475	25434	0.297
22515	0.395	23235	0.387	23455	0.039	24225	0.381	24445	0.185	25215	0.385	25435	0.206
22521	0.582	23241	0.557	23511	0.667	24231	0.644	24451	0.296	25221	0.572	25441	0.376
22522	0.495	23242	0.470	23512	0.580	24232	0.556	24452	0.209	25222	0.484	25442	0.289
22523	0.472	23243	0.447	23513	0.557	24233	0.534	24453	0.186	25223	0.462	25443	0.266
22524	0.385	23244	0.359	23514	0.469	24234	0.446	24454	0.099	25224	0.374	25444	0.179
22525	0.294	23245	0.269	23515	0.379	24235	0.356	24455	0.008	25225	0.284	25445	0.088
22531	0.557	23251	0.380	23521	0.566	24241	0.526	24511	0.636	25231	0.547	25451	0.199
22532	0.470	23252	0.292	23522	0.479	24242	0.438	24512	0.548	25232	0.459	25452	0.112
22533	0.447	23253	0.270	23523	0.456	24243	0.416	24513	0.526	25233	0.437	25453	0.089
22534	0.360	23254	0.182	23524	0.369	24244	0.328	24514	0.438	25234	0.349	25454	0.002
22535	0.269	23255	0.092	23525	0.278	24245	0.238	24515	0.348	25235	0.259	25455	– 0.089
22541	0.439	23311	0.788	23531	0.541	24251	0.349	24521	0.535	25241	0.429	25511	0.539
22542	0.352	23312	0.701	23532	0.454	24252	0.261	24522	0.447	25242	0.342	25512	0.452
22543	0.329	23313	0.678	23533	0.431	24253	0.239	24523	0.425	25243	0.319	25513	0.429
22544	0.242	23314	0.590	23534	0.343	24254	0.151	24524	0.337	25244	0.231	25514	0.341
22545	0.151	23315	0.500	23535	0.253	24255	0.061	24525	0.247	25245	0.141	25515	0.251
22551	0.262	23321	0.687	23541	0.423	24311	0.757	24531	0.510	25251	0.252	25521	0.438
22552	0.175	23322	0.600	23542	0.336	24312	0.669	24532	0.422	25252	0.164	25522	0.351
22553	0.152	23323	0.577	23543	0.313	24313	0.647	24533	0.400	25253	0.142	25523	0.328
22554	0.065	23324	0.490	23544	0.226	24314	0.559	24534	0.312	25254	0.054	25524	0.241
22555	– 0.026	23325	0.399	23545	0.135	24315	0.469	24535	0.222	25255	– 0.036	25525	0.150
23111	0.853	23331	0.662	23551	0.246	24321	0.656	24541	0.392	25311	0.660	25531	0.413
23112	0.766	23332	0.575	23552	0.159	24322	0.568	24542	0.305	25312	0.573	25532	0.326
23113	0.743	23333	0.552	23553	0.136	24323	0.546	24543	0.282	25313	0.550	25533	0.303
23114	0.655	23334	0.464	23554	0.048	24324	0.458	24544	0.195	25314	0.462	25534	0.215
23115	0.565	23335	0.374	23555	– 0.042	24325	0.368	24545	0.104	25315	0.372	25535	0.125
23121	0.752	23341	0.544	24111	0.822	24331	0.631	24551	0.215	25321	0.559	25541	0.295
23122	0.665	23342	0.457	24112	0.734	24332	0.543	24552	0.127	25322	0.472	25542	0.208
23123	0.642	23343	0.434	24113	0.712	24333	0.521	24553	0.105	25323	0.449	25543	0.185
23124	0.555	23344	0.347	24114	0.624	24334	0.433	24554	0.017	25324	0.362	25544	0.098
23125	0.464	23345	0.256	24115	0.534	24335	0.343	24555	– 0.073	25325	0.271	25545	0.007
23131	0.727	23351	0.367	24121	0.721	24341	0.513	25111	0.725	25331	0.534	25551	0.118
23132	0.640	23352	0.280	24122	0.633	24342	0.426	25112	0.638	25332	0.447	25552	0.031
23133	0.617	23353	0.257	24123	0.611	24343	0.403	25113	0.615	25333	0.424	25553	0.008
23134	0.529	23354	0.169	24124	0.523	24344	0.316	25114	0.527	25334	0.336	25554	– 0.080
23135	0.439	23355	0.079	24125	0.433	24345	0.225	25115	0.437	25335	0.246	25555	– 0.170
23141	0.609	23411	0.748	24131	0.696	24351	0.336	25121	0.624	25341	0.416	31111	0.939
23142	0.522	23412	0.661	24132	0.608	24352	0.248	25122	0.537	25342	0.329	31112	0.852
23143	0.499	23413	0.638	24133	0.586	24353	0.226	25123	0.514	25343	0.306	31113	0.829
23144	0.412	23414	0.551	24134	0.498	24354	0.138	25124	0.427	25344	0.219	31114	0.742
23145	0.321	23415	0.460	24135	0.408	24355	0.048	25125	0.336	25345	0.128	31115	0.651
23151	0.432	23421	0.647	24141	0.578	24411	0.717	25131	0.599	25351	0.239	31121	0.838
23152	0.345	23422	0.560	24142	0.491	24412	0.630	25132	0.512	25352	0.152	31122	0.751
23153	0.322	23423	0.537	24143	0.468	24413	0.607	25133	0.489	25353	0.129	31123	0.728
23154	0.234	23424	0.450	24144	0.381	24414	0.520	25134	0.401	25354	0.041	31124	0.641
23155	0.144	23425	0.359	24145	0.290	24415	0.429	25135	0.311	25355	– 0.049	31125	0.550
31131	0.813	31351	0.453	32121	0.757	32341	0.549	33111	0.842	33331	0.651	33551	0.235
31132	0.726	31352	0.366	32122	0.670	32342	0.462	33112	0.755	33332	0.564	33552	0.148
31133	0.703	31353	0.343	32123	0.647	32343	0.439	33113	0.732	33333	0.541	33553	0.125
31134	0.616	31354	0.256	32124	0.560	32344	0.352	33114	0.645	33334	0.454	33554	0.038
31135	0.525	31355	0.165	32125	0.469	32345	0.261	33115	0.554	33335	0.363	33555	– 0.053
31141	0.695	31411	0.834	32131	0.732	32351	0.372	33121	0.741	33341	0.533	34111	0.811
31142	0.608	31412	0.747	32132	0.645	32352	0.285	33122	0.654	33342	0.446	34112	0.724
31143	0.585	31413	0.724	32133	0.622	32353	0.262	33123	0.631	33343	0.423	34113	0.701
31144	0.498	31414	0.637	32134	0.535	32354	0.175	33124	0.544	33344	0.336	34114	0.614
31145	0.407	31415	0.546	32135	0.444	32355	0.084	33125	0.453	33345	0.245	34115	0.523
31151	0.518	31421	0.733	32141	0.614	32411	0.753	33131	0.716	33351	0.356	34121	0.710
31152	0.431	31422	0.646	32142	0.527	32412	0.666	33132	0.629	33352	0.269	34122	0.623
31153	0.408	31423	0.623	32143	0.504	32413	0.643	33133	0.606	33353	0.246	34123	0.600
31154	0.321	31424	0.536	32144	0.417	32414	0.556	33134	0.519	33354	0.159	34124	0.513
31155	0.230	31425	0.445	32145	0.326	32415	0.465	33135	0.428	33355	0.068	34125	0.422
31211	0.887	31431	0.708	32151	0.437	32421	0.653	33141	0.598	33411	0.737	34131	0.685
31212	0.800	31432	0.621	32152	0.350	32422	0.565	33142	0.511	33412	0.650	34132	0.598
31213	0.777	31433	0.598	32153	0.327	32423	0.543	33143	0.488	33413	0.627	34133	0.575
31214	0.690	31434	0.511	32154	0.240	32424	0.455	33144	0.401	33414	0.540	34134	0.488
31215	0.599	31435	0.420	32155	0.149	32425	0.365	33145	0.310	33415	0.449	34135	0.397
31221	0.786	31441	0.590	32211	0.806	32431	0.627	33151	0.421	33421	0.636	34141	0.567
31222	0.699	31442	0.503	32212	0.719	32432	0.540	33152	0.334	33422	0.549	34142	0.480
31223	0.676	31443	0.480	32213	0.696	32433	0.517	33153	0.311	33423	0.526	34143	0.457
31224	0.589	31444	0.393	32214	0.609	32434	0.430	33154	0.224	33424	0.439	34144	0.370
31225	0.498	31445	0.302	32215	0.518	32435	0.339	33155	0.133	33425	0.348	34145	0.279
31231	0.761	31451	0.413	32221	0.705	32441	0.510	33211	0.790	33431	0.611	34151	0.390
31232	0.674	31452	0.326	32222	0.618	32442	0.422	33212	0.703	33432	0.524	34152	0.303
31233	0.651	31453	0.303	32223	0.595	32443	0.400	33213	0.680	33433	0.501	34153	0.280
31234	0.564	31454	0.216	32224	0.508	32444	0.312	33214	0.593	33434	0.414	34154	0.193
31235	0.473	31455	0.125	32225	0.417	32445	0.222	33215	0.502	33435	0.323	34155	0.102
31241	0.643	31511	0.753	32231	0.680	32451	0.332	33221	0.689	33441	0.493	34211	0.759
31242	0.556	31512	0.666	32232	0.593	32452	0.245	33222	0.602	33442	0.406	34212	0.671
31243	0.533	31513	0.643	32233	0.570	32453	0.222	33223	0.579	33443	0.383	34213	0.649
31244	0.446	31514	0.556	32234	0.483	32454	0.135	33224	0.492	33444	0.296	34214	0.561
31245	0.355	31515	0.465	32235	0.392	32455	0.044	33225	0.401	33445	0.205	34215	0.471
31251	0.466	31521	0.652	32241	0.562	32511	0.672	33231	0.664	33451	0.316	34221	0.658
31252	0.379	31522	0.565	32242	0.475	32512	0.585	33232	0.577	33452	0.229	34222	0.571
31253	0.356	31523	0.542	32243	0.452	32513	0.562	33233	0.554	33453	0.206	34223	0.548
31254	0.269	31524	0.455	32244	0.365	32514	0.475	33234	0.467	33454	0.119	34224	0.460
31255	0.178	31525	0.364	32245	0.274	32515	0.384	33235	0.376	33455	0.028	34225	0.370
31311	0.874	31531	0.627	32251	0.385	32521	0.571	33241	0.546	33511	0.656	34231	0.633
31312	0.787	31532	0.540	32252	0.298	32522	0.484	33242	0.459	33512	0.569	34232	0.545
31313	0.764	31533	0.517	32253	0.275	32523	0.461	33243	0.436	33513	0.546	34233	0.523
31314	0.677	31534	0.430	32254	0.188	32524	0.374	33244	0.349	33514	0.459	34234	0.435
31315	0.586	31535	0.339	32255	0.097	32525	0.283	33245	0.258	33515	0.368	34235	0.345
31321	0.773	31541	0.509	32311	0.793	32531	0.546	33251	0.369	33521	0.555	34241	0.515
31322	0.686	31542	0.422	32312	0.706	32532	0.459	33252	0.282	33522	0.468	34242	0.428
31323	0.663	31543	0.399	32313	0.683	32533	0.436	33253	0.259	33523	0.445	34243	0.405
31324	0.576	31544	0.312	32314	0.596	32534	0.349	33254	0.172	33524	0.358	34244	0.318
31325	0.485	31545	0.221	32315	0.505	32535	0.258	33255	0.081	33525	0.267	34245	0.227
31331	0.748	31551	0.332	32321	0.692	32541	0.428	33311	0.777	33531	0.530	34251	0.338
31332	0.661	31552	0.245	32322	0.605	32542	0.341	33312	0.690	33532	0.443	34252	0.250
31333	0.638	31553	0.222	32323	0.582	32543	0.318	33313	0.667	33533	0.420	34253	0.228
31334	0.551	31554	0.135	32324	0.495	32544	0.231	33314	0.580	33534	0.333	34254	0.140
31335	0.460	31555	0.044	32325	0.404	32545	0.140	33315	0.489	33535	0.242	34255	0.050
31341	0.630	32111	0.858	32331	0.667	32551	0.251	33321	0.676	33541	0.412	34311	0.746
31342	0.543	32112	0.771	32332	0.580	32552	0.164	33322	0.589	33542	0.325	34312	0.659
31343	0.520	32113	0.748	32333	0.557	32553	0.141	33323	0.566	33543	0.302	34313	0.636
31344	0.433	32114	0.661	32334	0.470	32554	0.054	33324	0.479	33544	0.215	34314	0.549
31345	0.342	32115	0.570	32335	0.379	32555	– 0.037	33325	0.388	33545	0.124	34315	0.458
34321	0.645	34541	0.381	35311	0.649	35531	0.402	41251	0.439	41521	0.626	42241	0.536
34322	0.558	34542	0.294	35312	0.562	35532	0.315	41252	0.352	41522	0.538	42242	0.449
34323	0.535	34543	0.271	35313	0.539	35533	0.292	41253	0.329	41523	0.516	42243	0.426
34324	0.448	34544	0.184	35314	0.452	35534	0.205	41254	0.242	41524	0.428	42244	0.339
34325	0.357	34545	0.093	35315	0.361	35535	0.114	41255	0.151	41525	0.338	42245	0.248
34331	0.620	34551	0.204	35321	0.548	35541	0.284	41311	0.848	41531	0.601	42251	0.359
34332	0.533	34552	0.117	35322	0.461	35542	0.197	41312	0.760	41532	0.513	42252	0.271
34333	0.510	34553	0.094	35323	0.438	35543	0.174	41313	0.738	41533	0.491	42253	0.249
34334	0.423	34554	0.007	35324	0.351	35544	0.087	41314	0.650	41534	0.403	42254	0.161
34335	0.332	34555	– 0.084	35325	0.260	35545	– 0.004	41315	0.560	41535	0.313	42255	0.071
34341	0.502	35111	0.714	35331	0.523	35551	0.107	41321	0.747	41541	0.483	42311	0.767
34342	0.415	35112	0.627	35332	0.436	35552	0.020	41322	0.659	41542	0.395	42312	0.680
34343	0.392	35113	0.604	35333	0.413	35553	– 0.003	41323	0.637	41543	0.373	42313	0.657
34344	0.305	35114	0.517	35334	0.326	35554	– 0.090	41324	0.549	41544	0.285	42314	0.569
34345	0.214	35115	0.426	35335	0.235	35555	– 0.181	41325	0.459	41545	0.195	42315	0.479
34351	0.325	35121	0.613	35341	0.405	41111	0.913	41331	0.722	41551	0.306	42321	0.666
34352	0.238	35122	0.526	35342	0.318	41112	0.825	41332	0.634	41552	0.218	42322	0.579
34353	0.215	35123	0.503	35343	0.295	41113	0.803	41333	0.612	41553	0.196	42323	0.556
34354	0.128	35124	0.416	35344	0.208	41114	0.715	41334	0.524	41554	0.108	42324	0.469
34355	0.037	35125	0.325	35345	0.117	41115	0.625	41335	0.434	41555	0.018	42325	0.378
34411	0.706	35131	0.588	35351	0.228	41121	0.812	41341	0.604	42111	0.832	42331	0.641
34412	0.619	35132	0.501	35352	0.141	41122	0.724	41342	0.516	42112	0.745	42332	0.554
34413	0.596	35133	0.478	35353	0.118	41123	0.702	41343	0.494	42113	0.722	42333	0.531
34414	0.509	35134	0.391	35354	0.031	41124	0.614	41344	0.406	42114	0.634	42334	0.443
34415	0.418	35135	0.300	35355	– 0.060	41125	0.524	41345	0.316	42115	0.544	42335	0.353
34421	0.605	35141	0.470	35411	0.609	41131	0.787	41351	0.427	42121	0.731	42341	0.523
34422	0.518	35142	0.383	35412	0.522	41132	0.699	41352	0.339	42122	0.644	42342	0.436
34423	0.495	35143	0.360	35413	0.499	41133	0.677	41353	0.317	42123	0.621	42343	0.413
34424	0.408	35144	0.273	35414	0.412	41134	0.589	41354	0.229	42124	0.534	42344	0.326
34425	0.317	35145	0.182	35415	0.321	41135	0.499	41355	0.139	42125	0.443	42345	0.235
34431	0.580	35151	0.293	35421	0.508	41141	0.669	41411	0.808	42131	0.706	42351	0.346
34432	0.493	35152	0.206	35422	0.421	41142	0.581	41412	0.721	42132	0.619	42352	0.259
34433	0.470	35153	0.183	35423	0.398	41143	0.559	41413	0.698	42133	0.596	42353	0.236
34434	0.383	35154	0.096	35424	0.311	41144	0.471	41414	0.611	42134	0.508	42354	0.148
34435	0.292	35155	0.005	35425	0.220	41145	0.381	41415	0.520	42135	0.418	42355	0.058
34441	0.462	35211	0.662	35431	0.483	41151	0.492	41421	0.707	42141	0.588	42411	0.727
34442	0.375	35212	0.575	35432	0.396	41152	0.404	41422	0.620	42142	0.501	42412	0.640
34443	0.352	35213	0.552	35433	0.373	41153	0.382	41423	0.597	42143	0.478	42413	0.617
34444	0.265	35214	0.465	35434	0.286	41154	0.294	41424	0.510	42144	0.391	42414	0.530
34445	0.174	35215	0.374	35435	0.195	41155	0.204	41425	0.419	42145	0.300	42415	0.439
34451	0.285	35221	0.561	35441	0.365	41211	0.860	41431	0.682	42151	0.411	42421	0.626
34452	0.198	35222	0.474	35442	0.278	41212	0.773	41432	0.595	42152	0.324	42422	0.539
34453	0.175	35223	0.451	35443	0.255	41213	0.750	41433	0.572	42153	0.301	42423	0.516
34454	0.088	35224	0.364	35444	0.168	41214	0.663	41434	0.485	42154	0.213	42424	0.429
34455	– 0.003	35225	0.273	35445	0.077	41215	0.572	41435	0.394	42155	0.123	42425	0.338
34511	0.625	35231	0.536	35451	0.188	41221	0.760	41441	0.564	42211	0.780	42431	0.601
34512	0.538	35232	0.449	35452	0.101	41222	0.672	41442	0.477	42212	0.692	42432	0.514
34513	0.515	35233	0.426	35453	0.078	41223	0.650	41443	0.454	42213	0.670	42433	0.491
34514	0.428	35234	0.339	35454	– 0.009	41224	0.562	41444	0.367	42214	0.582	42434	0.404
34515	0.337	35235	0.248	35455	– 0.100	41225	0.472	41445	0.276	42215	0.492	42435	0.313
34521	0.524	35241	0.418	35511	0.528	41231	0.734	41451	0.387	42221	0.679	42441	0.483
34522	0.437	35242	0.331	35512	0.441	41232	0.647	41452	0.300	42222	0.591	42442	0.396
34523	0.414	35243	0.308	35513	0.418	41233	0.624	41453	0.277	42223	0.569	42443	0.373
34524	0.327	35244	0.221	35514	0.331	41234	0.537	41454	0.190	42224	0.481	42444	0.286
34525	0.236	35245	0.130	35515	0.240	41235	0.446	41455	0.099	42225	0.391	42445	0.195
34531	0.499	35251	0.241	35521	0.427	41241	0.617	41511	0.727	42231	0.654	42451	0.306
34532	0.412	35252	0.154	35522	0.340	41242	0.529	41512	0.639	42232	0.566	42452	0.219
34533	0.389	35253	0.131	35523	0.317	41243	0.507	41513	0.617	42233	0.544	42453	0.196
34534	0.302	35254	0.044	35524	0.230	41244	0.419	41514	0.529	42234	0.456	42454	0.109
34535	0.211	35255	– 0.047	35525	0.139	41245	0.329	41515	0.439	42235	0.366	42455	0.018
42511	0.646	43231	0.637	43451	0.290	44221	0.631	44441	0.436	45211	0.635	45431	0.457
42512	0.559	43232	0.550	43452	0.203	44222	0.544	44442	0.349	45212	0.548	45432	0.370
42513	0.536	43233	0.527	43453	0.180	44223	0.521	44443	0.326	45213	0.525	45433	0.347
42514	0.448	43234	0.440	43454	0.093	44224	0.434	44444	0.239	45214	0.438	45434	0.260
42515	0.358	43235	0.349	43455	0.002	44225	0.343	44445	0.148	45215	0.347	45435	0.169
42521	0.545	43241	0.520	43511	0.630	44231	0.606	44451	0.259	45221	0.535	45441	0.339
42522	0.458	43242	0.432	43512	0.542	44232	0.519	44452	0.171	45222	0.447	45442	0.252
42523	0.435	43243	0.410	43513	0.520	44233	0.496	44453	0.149	45223	0.425	45443	0.229
42524	0.348	43244	0.322	43514	0.432	44234	0.409	44454	0.061	45224	0.337	45444	0.142
42525	0.257	43245	0.232	43515	0.342	44235	0.318	44455	– 0.029	45225	0.247	45445	0.051
42531	0.520	43251	0.342	43521	0.529	44241	0.489	44511	0.598	45231	0.509	45451	0.162
42532	0.433	43252	0.255	43522	0.441	44242	0.401	44512	0.511	45232	0.422	45452	0.075
42533	0.410	43253	0.232	43523	0.419	44243	0.379	44513	0.488	45233	0.399	45453	0.052
42534	0.322	43254	0.145	43524	0.331	44244	0.291	44514	0.401	45234	0.312	45454	– 0.035
42535	0.232	43255	0.054	43525	0.241	44245	0.201	44515	0.310	45235	0.221	45455	– 0.126
42541	0.402	43311	0.751	43531	0.504	44251	0.311	44521	0.498	45241	0.392	45511	0.502
42542	0.315	43312	0.663	43532	0.416	44252	0.224	44522	0.410	45242	0.304	45512	0.414
42543	0.292	43313	0.641	43533	0.394	44253	0.201	44523	0.388	45243	0.282	45513	0.392
42544	0.205	43314	0.553	43534	0.306	44254	0.114	44524	0.300	45244	0.194	45514	0.304
42545	0.114	43315	0.463	43535	0.216	44255	0.023	44525	0.210	45245	0.104	45515	0.214
42551	0.225	43321	0.650	43541	0.386	44311	0.719	44531	0.472	45251	0.214	45521	0.401
42552	0.138	43322	0.562	43542	0.298	44312	0.632	44532	0.385	45252	0.127	45522	0.313
42553	0.115	43323	0.540	43543	0.276	44313	0.609	44533	0.362	45253	0.104	45523	0.291
42554	0.027	43324	0.452	43544	0.188	44314	0.522	44534	0.275	45254	0.017	45524	0.203
42555	– 0.063	43325	0.362	43545	0.098	44315	0.431	44535	0.184	45255	– 0.074	45525	0.113
43111	0.816	43331	0.625	43551	0.209	44321	0.619	44541	0.355	45311	0.623	45531	0.376
43112	0.728	43332	0.537	43552	0.121	44322	0.531	44542	0.267	45312	0.535	45532	0.288
43113	0.706	43333	0.515	43553	0.099	44323	0.509	44543	0.245	45313	0.513	45533	0.266
43114	0.618	43334	0.427	43554	0.011	44324	0.421	44544	0.157	45314	0.425	45534	0.178
43115	0.528	43335	0.337	43555	– 0.079	44325	0.331	44545	0.067	45315	0.335	45535	0.088
43121	0.715	43341	0.507	44111	0.784	44331	0.593	44551	0.177	45321	0.522	45541	0.258
43122	0.627	43342	0.419	44112	0.697	44332	0.506	44552	0.090	45322	0.434	45542	0.170
43123	0.605	43343	0.397	44113	0.674	44333	0.483	44553	0.067	45323	0.412	45543	0.148
43124	0.517	43344	0.309	44114	0.587	44334	0.396	44554	– 0.020	45324	0.324	45544	0.060
43125	0.427	43345	0.219	44115	0.496	44335	0.305	44555	– 0.111	45325	0.234	45545	– 0.030
43131	0.690	43351	0.330	44121	0.684	44341	0.476	45111	0.688	45331	0.497	45551	0.081
43132	0.602	43352	0.242	44122	0.596	44342	0.388	45112	0.600	45332	0.409	45552	– 0.007
43133	0.580	43353	0.220	44123	0.574	44343	0.366	45113	0.578	45333	0.387	45553	– 0.029
43134	0.492	43354	0.132	44124	0.486	44344	0.278	45114	0.490	45334	0.299	45554	– 0.117
43135	0.402	43355	0.042	44125	0.396	44345	0.188	45115	0.400	45335	0.209	45555	– 0.207
43141	0.572	43411	0.711	44131	0.658	44351	0.298	45121	0.587	45341	0.379	51111	0.573
43142	0.484	43412	0.624	44132	0.571	44352	0.211	45122	0.499	45342	0.291	51112	0.486
43143	0.462	43413	0.601	44133	0.548	44353	0.188	45123	0.477	45343	0.269	51113	0.463
43144	0.374	43414	0.514	44134	0.461	44354	0.101	45124	0.389	45344	0.181	51114	0.376
43145	0.284	43415	0.423	44135	0.370	44355	0.010	45125	0.299	45345	0.091	51115	0.285
43151	0.395	43421	0.610	44141	0.541	44411	0.680	45131	0.562	45351	0.202	51121	0.472
43152	0.307	43422	0.523	44142	0.453	44412	0.592	45132	0.474	45352	0.114	51122	0.385
43153	0.285	43423	0.500	44143	0.431	44413	0.570	45133	0.452	45353	0.092	51123	0.362
43154	0.197	43424	0.413	44144	0.343	44414	0.482	45134	0.364	45354	0.004	51124	0.275
43155	0.107	43425	0.322	44145	0.253	44415	0.392	45135	0.274	45355	– 0.086	51125	0.184
43211	0.763	43431	0.585	44151	0.363	44421	0.579	45141	0.444	45411	0.583	51131	0.447
43212	0.676	43432	0.498	44152	0.276	44422	0.492	45142	0.356	45412	0.496	51132	0.360
43213	0.653	43433	0.475	44153	0.253	44423	0.469	45143	0.334	45413	0.473	51133	0.337
43214	0.566	43434	0.388	44154	0.166	44424	0.381	45144	0.246	45414	0.386	51134	0.250
43215	0.475	43435	0.297	44155	0.075	44425	0.291	45145	0.156	45415	0.295	51135	0.159
43221	0.663	43441	0.467	44211	0.732	44431	0.554	45151	0.267	45421	0.482	51141	0.329
43222	0.575	43442	0.380	44212	0.645	44432	0.466	45152	0.179	45422	0.395	51142	0.242
43223	0.553	43443	0.357	44213	0.622	44433	0.444	45153	0.157	45423	0.372	51143	0.219
43224	0.465	43444	0.270	44214	0.535	44434	0.356	45154	0.069	45424	0.285	51144	0.132
43225	0.375	43445	0.179	44215	0.444	44435	0.266	45155	– 0.021	45425	0.194	51145	0.041
51151	0.152	51421	0.367	52141	0.248	52411	0.387	53131	0.350	53351	– 0.010	54121	0.344
51152	0.065	51422	0.280	52142	0.161	52412	0.300	53132	0.263	53352	– 0.097	54122	0.257
51153	0.042	51423	0.257	52143	0.138	52413	0.277	53133	0.240	53353	– 0.120	54123	0.234
51154	– 0.045	51424	0.170	52144	0.051	52414	0.190	53134	0.153	53354	– 0.207	54124	0.147
51155	– 0.136	51425	0.079	52145	– 0.040	52415	0.099	53135	0.062	53355	– 0.298	54125	0.056
51211	0.521	51431	0.342	52151	0.071	52421	0.287	53141	0.232	53411	0.371	54131	0.319
51212	0.434	51432	0.255	52152	– 0.016	52422	0.199	53142	0.145	53412	0.284	54132	0.232
51213	0.411	51433	0.232	52153	– 0.039	52423	0.177	53143	0.122	53413	0.261	54133	0.209
51214	0.324	51434	0.145	52154	– 0.126	52424	0.089	53144	0.035	53414	0.174	54134	0.122
51215	0.233	51435	0.054	52155	– 0.217	52425	– 0.001	53145	– 0.056	53415	0.083	54135	0.031
51221	0.420	51441	0.224	52211	0.440	52431	0.261	53151	0.055	53421	0.270	54141	0.201
51222	0.333	51442	0.137	52212	0.353	52432	0.174	53152	– 0.032	53422	0.183	54142	0.114
51223	0.310	51443	0.114	52213	0.330	52433	0.151	53153	– 0.055	53423	0.160	54143	0.091
51224	0.223	51444	0.027	52214	0.243	52434	0.064	53154	– 0.142	53424	0.073	54144	0.004
51225	0.132	51445	– 0.064	52215	0.152	52435	– 0.027	53155	– 0.233	53425	– 0.018	54145	– 0.087
51231	0.395	51451	0.047	52221	0.339	52441	0.144	53211	0.424	53431	0.245	54151	0.024
51232	0.308	51452	– 0.040	52222	0.252	52442	0.056	53212	0.337	53432	0.158	54152	– 0.063
51233	0.285	51453	– 0.063	52223	0.229	52443	0.034	53213	0.314	53433	0.135	54153	– 0.086
51234	0.198	51454	– 0.150	52224	0.142	52444	– 0.054	53214	0.227	53434	0.048	54154	– 0.173
51235	0.107	51455	– 0.241	52225	0.051	52445	– 0.144	53215	0.136	53435	– 0.043	54155	– 0.264
51241	0.277	51511	0.387	52231	0.314	52451	– 0.034	53221	0.323	53441	0.127	54211	0.393
51242	0.190	51512	0.300	52232	0.227	52452	– 0.121	53222	0.236	53442	0.040	54212	0.305
51243	0.167	51513	0.277	52233	0.204	52453	– 0.144	53223	0.213	53443	0.017	54213	0.283
51244	0.080	51514	0.190	52234	0.117	52454	– 0.231	53224	0.126	53444	– 0.070	54214	0.195
51245	– 0.011	51515	0.099	52235	0.026	52455	– 0.322	53225	0.035	53445	– 0.161	54215	0.105
51251	0.100	51521	0.286	52241	0.196	52511	0.306	53231	0.298	53451	– 0.050	54221	0.292
51252	0.013	51522	0.199	52242	0.109	52512	0.219	53232	0.211	53452	– 0.137	54222	0.205
51253	– 0.010	51523	0.176	52243	0.086	52513	0.196	53233	0.188	53453	– 0.160	54223	0.182
51254	– 0.097	51524	0.089	52244	– 0.001	52514	0.109	53234	0.101	53454	– 0.247	54224	0.094
51255	– 0.188	51525	– 0.002	52245	– 0.092	52515	0.018	53235	0.010	53455	– 0.338	54225	0.004
51311	0.508	51531	0.261	52251	0.019	52521	0.205	53241	0.180	53511	0.290	54231	0.267
51312	0.421	51532	0.174	52252	– 0.068	52522	0.118	53242	0.093	53512	0.203	54232	0.179
51313	0.398	51533	0.151	52253	– 0.091	52523	0.095	53243	0.070	53513	0.180	54233	0.157
51314	0.311	51534	0.064	52254	– 0.178	52524	0.008	53244	– 0.017	53514	0.093	54234	0.069
51315	0.220	51535	– 0.027	52255	– 0.269	52525	– 0.083	53245	– 0.108	53515	0.002	54235	– 0.021
51321	0.407	51541	0.143	52311	0.427	52531	0.180	53251	0.003	53521	0.189	54241	0.149
51322	0.320	51542	0.056	52312	0.340	52532	0.093	53252	– 0.084	53522	0.102	54242	0.062
51323	0.297	51543	0.033	52313	0.317	52533	0.070	53253	– 0.107	53523	0.079	54243	0.039
51324	0.210	51544	– 0.054	52314	0.230	52534	– 0.017	53254	– 0.194	53524	– 0.008	54244	– 0.048
51325	0.119	51545	– 0.145	52315	0.139	52535	– 0.108	53255	– 0.285	53525	– 0.099	54245	– 0.139
51331	0.382	51551	– 0.034	52321	0.326	52541	0.062	53311	0.411	53531	0.164	54251	– 0.028
51332	0.295	51552	– 0.121	52322	0.239	52542	– 0.025	53312	0.324	53532	0.077	54252	– 0.116
51333	0.272	51553	– 0.144	52323	0.216	52543	– 0.048	53313	0.301	53533	0.054	54253	– 0.138
51334	0.185	51554	– 0.231	52324	0.129	52544	– 0.135	53314	0.214	53534	– 0.033	54254	– 0.226
51335	0.094	51555	– 0.322	52325	0.038	52545	– 0.226	53315	0.123	53535	– 0.124	54255	– 0.316
51341	0.264	52111	0.492	52331	0.301	52551	– 0.115	53321	0.310	53541	0.046	54311	0.380
51342	0.177	52112	0.405	52332	0.214	52552	– 0.202	53322	0.223	53542	– 0.041	54312	0.293
51343	0.154	52113	0.382	52333	0.191	52553	– 0.225	53323	0.200	53543	– 0.064	54313	0.270
51344	0.067	52114	0.295	52334	0.104	52554	– 0.312	53324	0.113	53544	– 0.151	54314	0.183
51345	– 0.024	52115	0.204	52335	0.013	52555	– 0.403	53325	0.022	53545	– 0.242	54315	0.092
51351	0.087	52121	0.391	52341	0.183	53111	0.476	53331	0.285	53551	– 0.131	54321	0.279
51352	0.000	52122	0.304	52342	0.096	53112	0.389	53332	0.198	53552	– 0.218	54322	0.192
51353	– 0.023	52123	0.281	52343	0.073	53113	0.366	53333	0.175	53553	– 0.241	54323	0.169
51354	– 0.110	52124	0.194	52344	– 0.014	53114	0.279	53334	0.088	53554	– 0.328	54324	0.082
51355	– 0.201	52125	0.103	52345	– 0.105	53115	0.188	53335	– 0.003	53555	– 0.419	54325	– 0.009
51411	0.468	52131	0.366	52351	0.006	53121	0.375	53341	0.167	54111	0.445	54331	0.254
51412	0.381	52132	0.279	52352	– 0.081	53122	0.288	53342	0.080	54112	0.358	54332	0.167
51413	0.358	52133	0.256	52353	– 0.104	53123	0.265	53343	0.057	54113	0.335	54333	0.144
51414	0.271	52134	0.169	52354	– 0.191	53124	0.178	53344	– 0.030	54114	0.248	54334	0.057
51415	0.180	52135	0.078	52355	– 0.282	53125	0.087	53345	– 0.121	54115	0.157	54335	– 0.034

State	Value	State	Value	State	Value	State	Value
54341	0.136	55111	0.348	55331	0.157	55551	– 0.259
54342	0.049	55112	0.261	55332	0.070	55552	– 0.346
54343	0.026	55113	0.238	55333	0.047	55553	– 0.369
54344	– 0.061	55114	0.151	55334	– 0.040	55554	– 0.456
54345	– 0.152	55115	0.060	55335	– 0.131	55555	– 0.547
54351	– 0.041	55121	0.247	55341	0.039		
54352	– 0.128	55122	0.160	55342	– 0.048		
54353	– 0.151	55123	0.137	55343	– 0.071		
54354	– 0.238	55124	0.050	55344	– 0.158		
54355	– 0.329	55125	– 0.041	55345	– 0.249		
54411	0.340	55131	0.222	55351	– 0.138		
54412	0.253	55132	0.135	55352	– 0.225		
54413	0.230	55133	0.112	55353	– 0.248		
54414	0.143	55134	0.025	55354	– 0.335		
54415	0.052	55135	– 0.066	55355	– 0.426		
54421	0.239	55141	0.104	55411	0.243		
54422	0.152	55142	0.017	55412	0.156		
54423	0.129	55143	– 0.006	55413	0.133		
54424	0.042	55144	– 0.093	55414	0.046		
54425	– 0.049	55145	– 0.184	55415	– 0.045		
54431	0.214	55151	– 0.073	55421	0.142		
54432	0.127	55152	– 0.160	55422	0.055		
54433	0.104	55153	– 0.183	55423	0.032		
54434	0.017	55154	– 0.270	55424	– 0.055		
54435	– 0.074	55155	– 0.361	55425	– 0.146		
54441	0.096	55211	0.296	55431	0.117		
54442	0.009	55212	0.209	55432	0.030		
54443	– 0.014	55213	0.186	55433	0.007		
54444	– 0.101	55214	0.099	55434	– 0.080		
54445	– 0.192	55215	0.008	55435	– 0.171		
54451	– 0.081	55221	0.195	55441	– 0.001		
54452	– 0.168	55222	0.108	55442	– 0.088		
54453	– 0.191	55223	0.085	55443	– 0.111		
54454	– 0.278	55224	– 0.002	55444	– 0.198		
54455	– 0.369	55225	– 0.093	55445	– 0.289		
54511	0.259	55231	0.170	55451	– 0.178		
54512	0.172	55232	0.083	55452	– 0.265		
54513	0.149	55233	0.060	55453	– 0.288		
54514	0.062	55234	– 0.027	55454	– 0.375		
54515	– 0.029	55235	– 0.118	55455	– 0.466		
54521	0.158	55241	0.052	55511	0.162		
54522	0.071	55242	– 0.035	55512	0.075		
54523	0.048	55243	– 0.058	55513	0.052		
54524	– 0.039	55244	– 0.145	55514	– 0.035		
54525	– 0.130	55245	– 0.236	55515	– 0.126		
54531	0.133	55251	– 0.125	55521	0.061		
54532	0.046	55252	– 0.212	55522	– 0.026		
54533	0.023	55253	– 0.235	55523	– 0.049		
54534	– 0.064	55254	– 0.322	55524	– 0.136		
54535	– 0.155	55255	– 0.413	55525	– 0.227		
54541	0.015	55311	0.283	55531	0.036		
54542	– 0.072	55312	0.196	55532	– 0.051		
54543	– 0.095	55313	0.173	55533	– 0.074		
54544	– 0.182	55314	0.086	55534	– 0.161		
54545	– 0.273	55315	– 0.005	55535	– 0.252		
54551	– 0.162	55321	0.182	55541	– 0.082		
54552	– 0.249	55322	0.095	55542	– 0.169		
54553	– 0.272	55323	0.072	55543	– 0.192		
54554	– 0.359	55324	– 0.015	55544	– 0.279		
54555	– 0.450	55325	– 0.106	55545	– 0.370		
